# Water quality dynamics in urban river of the semiarid region under anthropogenic pressure: a spatiotemporal analysis in the stretch of the municipality of Mossoró (Brazil)

**DOI:** 10.1007/s10661-026-15476-x

**Published:** 2026-05-28

**Authors:** Adler Lincoln Severiano da Silva, Hélio Nogueira Bezerra, Phâmella Kalliny Pereira Farias, Maria Sara Martins da Silva, Leandra Karla Oliveira Costa, Letícia Vitória Melo da Silva, Carla Natanieli de Oliveira Batista, Daniela da Costa Leite Coelho, Maria Valdiglezia de Mesquita Arruda, Luiz Fernando de Sousa Antunes, Rafael Oliveira Batista

**Affiliations:** Center of Agrarian Sciences, Federal Rural University of the Semi-Arid, Mossoró, Rio Grande do Norte 59625-900 Brazil

**Keywords:** Surface water body, Water pollution, Monitoring, Sustainability index

## Abstract

Over the years, urban rivers have suffered from environmental degradation due to anthropogenic activities, becoming a public concern. Thus, the objective of this study was to evaluate the spatiotemporal evolution of water quality in the Apodi-Mossoró River, in the urban section of Mossoró-RN, during the dry and rainy seasons. To this end, the physical–chemical and microbiological attributes of water samples collected at six points along the urban stretch of the Apodi-Mossoró River were characterized. Using the water quality data, the Water Quality Index was calculated, and multivariate statistical analysis was performed. The Water Quality Index varied considerably. At the Genésio Dam, a more positive assessment was observed, with ratings ranging from excellent to good. However, the other points showed significant variations, especially the Barrocas Dam, which received all ratings. The attributes that most influenced the results were total Kjeldahl nitrogen, turbidity and biochemical oxygen demand. The river water showed a high salt content, resulting in a classification as brackish at all points evaluated for most of the year, except during the rainy season, when it was considered fresh.

## Highlights

- The water quality of the Apodi-Mossoró River is directly influenced by climate and urbanization.
- The WQI varies from excellent to poor, covering all classifications according to CETESB parameters.
- During the rainy season, the water of the Apodi-Mossoró River changes from brackish to fresh throughout the urban stretch of Mossoró.
- TKN, TURB, and BOD, are the most influential attributes in the WQI classification of the Apodi-Mossoró River.
- Water quality declines downstream due to anthropic activity and increased pollutants.

**Graphical Abstract:**

## Graphical abstract

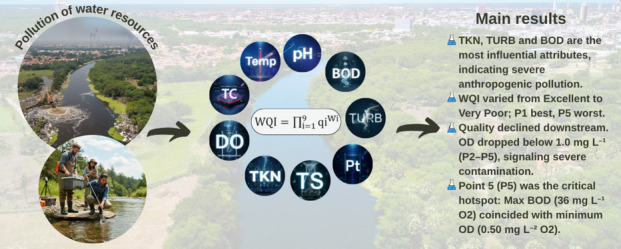

## Introduction

Surface water comprises all liquid water present on the Earth’s surface, including both ocean waters and those stored in continental reservoirs, such as streams, creeks, rivers, brooks, lakes, swamps, and artificial reservoirs. On a global scale, liquid surface water accounts for more than 97% of the Earth’s hydrosphere, with approximately 96% consisting of saltwater from the oceans. In contrast, only about 1.1% corresponds to freshwater, of which approximately 99% is stored in underground aquifers, while only 1% is available in the form of surface freshwater (Saeed et al., 2022; Syeed et al., 2023).

In this context, the growing global water scarcity has been intensified by multiple interrelated factors, including prolonged droughts, increased anthropogenic demand, pollution of water bodies, deforestation, and inefficient management of natural resources. Therefore, it is estimated that approximately 30% of the global population faces water scarcity conditions, compromising environmental sustainability, water security, and socioeconomic development in various regions of the planet (Van Vliet et al., 2021).

Surface freshwater plays a fundamental role not only in the complex dynamics of the global hydrological cycle and the functioning of terrestrial ecosystems, but also due to the high human dependence on this natural resource for multiple uses, including domestic supply, transportation, agricultural production, and energy generation. However, the quality of these waters is continuously influenced by a combination of natural factors, such as atmospheric, climatic, hydrodynamic, lithological, and topographic elements, and anthropogenic factors, associated with urbanization, industrialization, mining, agricultural activities, changes in land use and occupation, in addition to the increasing presence of emerging contaminants in aquatic environments (Prasad et al., 2022; Singh et al., 2024; Uddin et al., 2021).

Thus, the degradation of water quality is one of the main challenges for the sustainable management of water resources on a global scale, especially in semiarid regions, where water scarcity manifests itself more intensely and frequently. Despite the relevance of this scenario, there is still limited scientific attention focused on characterizing water quality and identifying critical pollutants in rivers located in semiarid environments. Furthermore, there is a lack of robust and effective methods for assessing water quality in these regions, which are particularly vulnerable to climatic and anthropogenic pressures (Sang et al., 2026).

Therefore, the deterioration of water quality constitutes a global-scale problem that directly compromises water availability and represents serious risks to human health. Contaminated water remains one of the main threats to humanity due to its high potential for spreading diseases and increasing associated mortality, especially from waterborne illnesses. Additionally, another highly relevant factor contributing to the degradation of water bodies is the direct discharge of untreated wastewater into lotic aquatic environments. The continuous introduction of these wastes promotes significant changes in ecosystems, since the pollutants present in the water can compromise the ecological balance of organisms and their natural habitats. Consequently, there is an intensification of environmental degradation processes, including the reduction of biodiversity and the impairment of essential ecological functions of aquatic ecosystems (Chaudhry & Malik, 2017; Ferreira et al., 2016; Wear et al., 2021).

Water quality assessment is essential for the sustainable management of water resources, as it is a fundamental tool for identifying the degree of pollution in water bodies and supporting the adoption of mitigating measures aimed at reducing the environmental impacts caused by pollutants, enhancing environmental, social, and governance (ESG) conditions. This process involves the integration of physical, chemical, and microbiological parameters, which must be compared to the limits established by current technical standards and legislation. Furthermore, for water quality monitoring to be effective, it is essential to obtain a comprehensive set of data, allowing for the correlation of multiple attributes and the identification of the main factors responsible for the characterization and variation of water quality. In this context, the determination of the Water Quality Index (WQI) stands out as an efficient and widely used approach for environmental monitoring, enabling the integrated assessment of water quality at different sampling points and over time, based on the combination of various water quality parameters. Thus, the WQI constitutes a relevant tool to support management strategies focused on the circular economy of water, evidence-based decision-making, and the strengthening of ESG indicators related to the sustainability of water resources (Dutta et al., 2018; Gad et al., 2022; Jiang et al., 2020; Melo et al., 2020).

Despite growing global concern about the degradation of water resources in semiarid regions, studies that comprehensively assess the spatio-temporal variability of the quality of lotic environments in the Brazilian semiarid region under the simultaneous influence of anthropogenic and geogenic pollution sources are still scarce, restricting the development of more efficient strategies for monitoring, conservation, and sustainable management of water resources. Therefore, due to the great importance of analyzing water resources and creating conservation strategies, the objective of this study was to carry out a spatio-temporal assessment of the quality of the urban stretch of a river in a semiarid region of northeastern Brazil, characterizing physical, chemical, and microbiological parameters during the dry and rainy seasons under conditions of anthropogenic and geogenic pollution. The hypothesis of this study is: The water quality of the urban stretch of a river in the semiarid region of northeastern Brazil presents significant spatial and temporal variation between the dry and rainy seasons, being more deteriorated in points under greater anthropogenic influence, especially due to the discharge of domestic and agro-industrial effluents, causing a greater magnitude of physical, chemical and microbiological parameters and lower values of the Water Quality Index.

## Materials and methods

### Location and characterization of the study area

This study was conducted in the urban section of the Apodi-Mossoró River, in the municipality of Mossoró, Rio Grande do Norte, Brazil. The soils in the study area were classified according to the Brazilian Soil Classification System (SIBCS) as “Latossolo vermelho-amarelo,” “Argissolo vermelho-amarelo,” “Neossolos quartzoarênicos,” “Cambissolos háplicos,” and “Neossolos litólicos,” which correspond respectively to Ferralsols, Acrisols, Arenosols, Cambisols, and Leptosols in the World Reference Base for Soil Resources-WRB/FAO (Bezerra et al., 2013; Santos et al., 2025).

Rio Grande do Norte has 16 river basins distributed throughout its territory. The Apodi-Mossoró River basin is the second largest in the state, located in the western region, with an area of 14,276 km^2^. The Apodi-Mossoró River is 210 km long, with its source on the border between the states of Rio Grande do Norte and Paraíba, flowing into the Atlantic Ocean on the border between the municipalities of Areia Branca and Grossos, which belong to the Costa Branca region of Rio Grande do Norte (Silva & Camargo, 2022).

The Apodi-Mossoró River is considered the most important water resource in the western region of the state of Rio Grande do Norte, crossing several municipalities in the state, with Mossoró being the municipality with the highest population density. Mossoró has a territorial extension of 2099.334 km^2^, with 73.55 km^2^ of urbanized area and a population of 264,577 inhabitants, where the sanitary sewage index is 52.96%, according to the Brazilian Institute of Geography and Statistics – IBGE (2025). The water quality of the Apodi-Mossoró River is impacted by anthropogenic activities such as the discharge of domestic and agro-industrial effluents in the vicinity of the riverbed, resulting in pollution.

Sampling took place between July 2022 and April 2023, with eight water samples collected at each of the six points chosen for the study. The section chosen for sampling covered approximately 10 km, with the strategic choice of points based on observations made at the site. This selection considered criteria that encompassed areas with different levels of human activity.

**Fig. 1:**
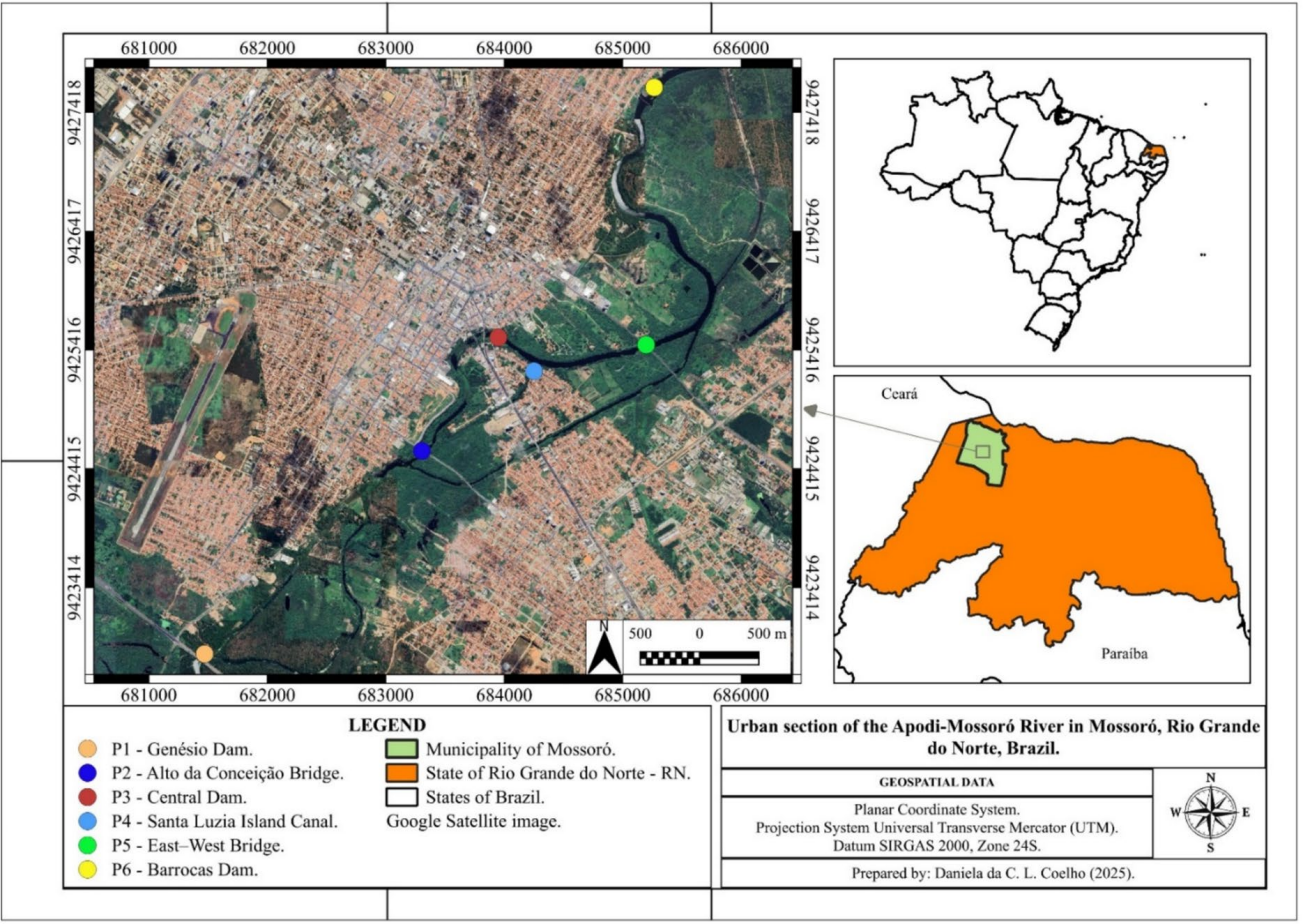
Image of the urban section of the Apodi-Mossoró River in Mossoró-RN, highlighting the six water sampling points

The first collection point (P1) was established at the Genésio Dam, located on the banks of the BR-304 highway (682,250 m E and 9,422,150 m S). The second point (P2) is located below the bridge on Avenida Coelho Neto, in the Alto da Conceição neighborhood (684,050 m E and 9,423,930 m S). The third point (P3) is located at the central dam in the city of Mossoró-RN (685,000 m E and 9,424,890 m S). The fourth point (P4) is in the Ilha de Santa Luzia neighborhood, a division of the river (685,300 m E and 9,424,610 m S). The fifth point (P5) is below the East–West Avenue bridge (686,240 m E and 9,424,840 m S) and the sixth and last point (P6) is centered on the Barrocas Dam (686,300 m E and 9,427,020 m S). It should be noted that the geographic coordinates are in the UTM/SIRGAS 2000 system in Zone 24S (Fig. 1).

### Climatic aspects

The municipality of Mossoró is located in the western part of the state of Rio Grande do Norte in the semiarid region (Silva & Camargo, 2022), where the climate is BSh, hot and dry, with a rainy season concentrated between May and July and intense drought from September to December. The city has an average air temperature of 26.5 °C and average annual precipitation of 794 mm (Alvares et al., 2013). The period from July 2022 to April 2023 was chosen to evaluate the physical, chemical, and microbiological attributes of the water in the urban section of the Apodi-Mossoró River during both the dry and rainy seasons.

**Fig. 2:**
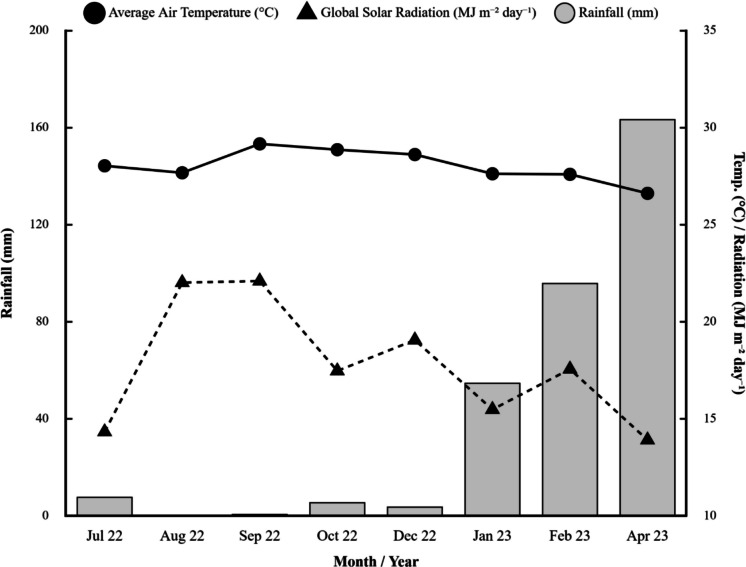
Monthly rainfall, accumulated global solar radiation, and average air temperature on the day of each water sample collection

During the sampling period, some climatic parameters were monitored, such as rainfall, global solar radiation and average air temperature, as shown in Fig. 2. These data were collected from the website https://siemu.ufersa.edu.br/dashboard, supplied by the Automatic Meteorological Station (EMA) of the Department of Engineering and Environmental Sciences (DECAM) of the Federal Rural University of the Semi-Arid Region (UFERSA), on the east campus of UFERSA Mossoró.

### Collection and analysis of water samples

The simple samples were collected using the method recommended by the Companhia Ambiental do Estado de São Paulo (CETESB, 2011), with 1.0 L polyethylene bottles immersed to a depth of 30 cm. They were then stored in an insulated box with ice at 4 °C until the physical–chemical analyses were performed. The samples for microbiological analysis were collected in sterile glass bottles. The analyses were performed in four laboratories at the Universidade Federal Rural do Semi-Árido (UFERSA) Mossoró-RN campus: (1) Soil, Water, and Plant Laboratory (LASAP); (2) Environmental Sanitation Laboratory (LASAN); (3) Semi-Arid Soil, Water, and Plant Analysis Laboratory (LASAPSA); and (4) Animal Product Inspection Laboratory (LIPOA).

The physical–chemical analyses were performed at LASAN and followed the criteria established in Standard Methods for Examination of Water and Wastewater (Lipps et al., 2023). Triplicate samples were taken and the averages of the repetitions were obtained. Table 1 shows the water quality variables analyzed, as well as the test methodology used.

**Table 1:** Water quality attributes analyzed at LASAN

Determination	Unit	Analytical method	
		Name	Number
pH	–	Electrometric	4500 H +
Temperature (WT)	°C	Digital thermometer	2550
Conductivity (EC)	m mho cm^−1^or m S cm^−1^	Laboratory method	2510 B
Dissolved oxygen (DO)	mgO_2 _L^−1^	Optical probe method	4500-O H
Total solids (TS)	mg L^−1^	Total solids at 103—105 °C	2540 B
Turbidity (TURB)	UNT	Nephelometric	2130 B
Total phosphorus (Pt)	mg P L^−1^	Ascorbic acid method with preliminary digestion using the persulfate method	4500-P E
Total Kjeldahl nitrogen (TKN)	mg N L^−1^	Semi-Micro-Kjeldahl	4500-Norg C
		Titrimetric with Preliminary Distillation	4500-NH3 C and 4500-NH3 B
Biochemical oxygen demand (BOD)	mgO_2 _L^−1^	5-day BOD	5210 B

The analyses followed the methodologies presented in the Standard Methods for Examination of Water and Wastewater Lipps et al.(2023)

In LASAP and LASAPSA, K^+^ and Na^+^ determined by flame photometry; Ca^2+^ and Mg^2+^ quantified by complexometry with EDTA, using eriochrome black-T as an indicator for magnesium and calcon for calcium; and Cl^−^ obtained by titration with silver nitrate, using potassium chromate as an indicator. The analyses followed the technical recommendations of the Empresa Brasileira de Pesquisa Agropecuária (Parron et al., 2011).

The determination of thermotolerant coliforms (TC) was performed using the multiple tube method (9221 E) at LIPOA following the recommendations of the Standard Methods for Examination of Water and Wastewater (Lipps et al., 2023). On site, water temperature (TEMP) and dissolved oxygen (DO) were measured using a field probe. Using the attributes pH, turbidity (TURB), biochemical oxygen demand (BOD), total solids (TS), total phosphorus (Pt), TKN, TEMP, DO, and TC, the Water Quality Index (WQI) was calculated and classified, adapted by CETESB (2024), of the National Sanitation Foundation (NSF), according to Eqs. 1 and 2, respectively.

1 $$\mathrm{W}\mathrm{Q}\mathrm{I}=\prod_{\mathrm{i}=1}^{9}{\mathrm{q}\mathrm{i}}^{\mathrm{W}\mathrm{i}}$$

2 $$\sum_{\mathrm{i}=1}^{9}\mathrm{W}\mathrm{i}=1$$

where WQI is the Water Quality Index, a number between 0 and 100; qi is the Quality of the *i*-th indicator, a number between 0 and 100, obtained from the respective equation of the “average quality variation curve,” depending on its concentration or measurement; wi is the weight corresponding to the *i*-th indicator, a number between 0 and 1, assigned according to its importance to the overall quality, decimal; and *i* is the indicator number, ranging from 1 to 9 (*n* = 9, i.e., the number of attributes that make up the WQI is 9).

The weights (wi) used in the calculation of WQI were as follows: DO (0.17), TC (0.15), pH (0.12), BOD (0.10), TKN (0.10), Pt (0.10), temperature deviation (0.10), TURB (0.08), and TS (0.08) (CETESB (2024b).

It should be noted that WQI uses total nitrogen (Nt) as one of the nine parameters in the equation. In this study, Nt was replaced by TKN due to the considerable contribution of organic and ammoniacal nitrogen from anthropogenic sources of point and diffuse pollution along the urban stretch of the Apodi-Mossoró River during the dry and rainy seasons.

The QualiGraf program, developed as an internal tool by the Department of Water Resources of the Fundação Cearense de Meteorologia e Recursos Hídricos (FUNCEME, 2023), was used to calculate the WQI.

In interpreting the attributes not used in the calculation of WQI, CONAMA Resolution No. 357/2005 (Brasil, 2005) was used as a comparative standard to assess the water quality of the urban stretch of the Apodi-Mossoró River in Mossoró-RN. It should be noted that for the purposes of classifying water as fresh, brackish, or saline, Article 42 of CONAMA (Brasil, 2005) was used, which establishes that for water bodies without classification, fresh waters (salinity ≤ 0.5 ‰) should be considered class 2, while brackish waters (0.5‰ < salinity < 30 0‰) and saline (salinity ≥ 30‰) should be considered class 1.

In assessing the salinity of water samples, EC was determined to estimate salinity based on the total dissolved solids (TDS) content, as described by Richards et al. (1954) and Rhoades et al. (1992) in Eqs. 3 and 4, respectively. This method correlates total dissolved solids with electrical conductivity, which is feasible because most dissolved substances are ions (Richter, 2009). To convert salinity from mg L^−1^ to parts per thousand (‰), divide the value by 1000.

For EC between 0.1 and 5.0 dS m^−1^, we have the following:

3 $$\mathrm{T}\mathrm{D}\mathrm{S}=640.\mathrm{E}\mathrm{C}$$

For EC greater than 5.0 dS m^−1^, we have the following:

4 $$\mathrm{T}\mathrm{D}\mathrm{S}=800.\mathrm{E}\mathrm{C}$$

where TDS is the salinity or total dissolved salts, in mg L^−1^, and EC is the electrical conductivity in water samples, in dS m^−1^.

### Statistical analysis

For river water salinity attributes (EC, K^+^, Na^+^, Ca^2+^, Mg^2+^, and Cl^−^), only descriptive statistics were used, employing mean, median, standard deviation, and coefficient of variation. Meanwhile, the WQI attributes (WT, pH, TURB, TS, DO, BOD, TC, TKN, and Pt) were subjected to more robust statistical procedures.

#### Descriptive statistics and outlier screening

The dataset comprises 48 observations (8 monthly campaigns × 6 sampling points) for 9 water quality variables from the WQI. For each variable, measures of central tendency (mean, median) and dispersion (standard deviation, coefficient of variation, minimum, maximum, first and third quartiles) were calculated. Possible outliers were assessed using boxplots and Tukey’s rule based on the interquartile range (IQR): observations below Q1 − 1.5·IQR or above Q3 + 1.5·IQR were flagged and cross-referenced with laboratory and field records (Hair Jr et al., 2019; Tukey, 1977). As all flagged values corresponded to environmentally plausible spatiotemporal variations (stormwater runoff events, organic matter peaks, and effluent discharges), no observations were removed. The median and coefficient of variation, robust to extreme values, were included to complement the description of the central tendency.

#### Normality assessment

Univariate normality was tested using the Shapiro–Wilk test (Razali & Wah, 2011), recommended for *n* < 50, at a significance level *α* = 0.05. The test rejected the normality hypothesis (*p* ≤ 0.05) for all nine variables in the raw data.

#### Data transformations and standardization

Variables with strongly right-skewed distributions and high coefficients of variation (TURB, DO, BOD, TC, TKN, Pt) were transformed by log10(x + 1) before multivariate analysis. Water temperature (WT) and pH were not transformed because they are not concentration/count variables, and pH is already expressed on a logarithmic scale. Total solids (TS) were also kept on the original scale because the log transformation worsened their normality. Preprocessing reduced skewness and improved the distributional behavior of several variables, although not all achieved normality after transformation: DO and TKN became normal (*p* > 0.05), while TURB, BOD, TC, and Pt remained non-normal, but with substantially reduced skewness. Subsequently, all variables (transformed when applicable; original in other cases) were standardized using z-scores (mean = 0; standard deviation = 1) before Pearson correlation analysis, PCA, and FA, to remove the influence of heterogeneous units of measurement.

#### Multivariate analysis

Multivariate analyses were performed in R version 4.6.0 (R Core Team, 2026), following the recommendations of Vicini et al. (2018) and Hair Jr et al. (2019). Linear associations between pre-processed and standardized variables were quantified using Pearson’s correlation matrix, with significance assessed at *α* = 0.05 (Callegari-Jacques, 2003: weak 0.00 <|*r*|< 0.30; moderate 0.30 ≤|*r*|< 0.60; strong 0.60 ≤|*r*|< 0.90; very strong 0.90 ≤|*r*|< 1.00).

Principal component analysis (PCA) was performed on the Pearson correlation matrix of the pre-processed and standardized variables (Jolliffe & Cadima, 2016). PCA is an orthogonal linear decomposition that produces uncorrelated components ordered by the total variance explained; it was used as an exploratory ordination technique for dimensionality reduction and identification of the main gradients of variation in water quality. Component retention followed Kaiser’s criterion (eigenvalues ≥ 1) (Oliveira Júnior et al., 2021; Chianca et al., 2023). Factor analysis (FA) was performed using the principal component method on the same correlation matrix, and a Varimax orthogonal rotation with Kaiser normalization (Kaiser, 1958) was applied to the retained components. Rotated loadings with |λ|≥ 0.65 were considered significant and strongly associated with the respective factor (Hair Jr et al., 2019). Sample adequacy was assessed using Bartlett’s test of sphericity and the Kaiser–Meyer–Olkin (KMO) index (Kaiser, 1974). Given the moderate sample size and limited overall KMO (0.48), PCA was treated as the primary multivariate method, and the solution rotated by Varimax was interpreted with caution.

## Results and discussion

### Water quality attributes related to salinity

Throughout the dry and rainy seasons, the salinity of the Apodi-Mossoró River varied according to the temporal factor (Table 2). Since this water body does not have a defined classification based on CONAMA Resolution No. 357/2005 (Brasil, 2005), the criteria established in article 42 of said resolution were adopted, where class 2 freshwater for salinity ≤ 0.5‰ and class 1 brackish water for salinity between 0.5‰ and 30‰. As established in the same resolution, during the dry season, the river was considered brackish water of class 1. During the rainy season, in the months of highest rainfall, the classification was changed to class 2 freshwater. This is important information for the classification of scarce lotic water bodies in the Brazilian semiarid region.

**Table 2:** Classification of water salinity at six points along the urban stretch of the river, using the thresholds of CONAMA Resolution No. 357/2005

Points	Period evaluated	Salinity (‰)	Classification	Points	Period evaluated	Salinity (‰)	Classification
P1	Jul22	0.53	Brackish	P4	July 2022	0.72	Brackish
	Aug22	0.69	Brackish		August 2022	0.83	Brackish
	Sep22	0.77	Brackish		September 2022	0.97	Brackish
	Oct22	0.97	Brackish		October 2022	1.14	Brackish
	Dec22	0.64	Brackish		December 2022	0.71	Brackish
	Jan23	0.66	Brackish		January 2023	0.61	Brackish
	Feb23	0.52	Brackish		February 2023	046	Fresh
	Apr23	0.13	Fresh		April 2023	0.10	Fresh
P2	Jul22	0.70	Brackish	P5	July 2022	0.74	Brackish
	Aug22	0.79	Brackish		August 2022	0.82	Brackish
	Sep22	0.92	Brackish		September 2022	0.93	Brackish
	Oct22	1.13	Brackish		October 2022	1.11	Brackish
	Dec22	0.68	Brackish		December 2022	0.94	Brackish
	Jan23	0.61	Brackish		January 2023	0.57	Brackish
	Feb23	0.48	Fresh		February 2023	0.45	Fresh
	Apr23	0.98	Brackish		April 2023	0.10	Fresh
P3	Jul22	0.74	Brackish	P6	July 2022	0.75	Brackish
	Aug22	0.82	Brackish		August 2022	0.81	Brackish
	Sep22	0.97	Brackish		September 2022	0.93	Brackish
	Oct22	1.06	Brackish		October 2022	1.08	Brackish
	Dec22	0.65	Brackish		December 2022	0.61	Brackish
	Jan23	0.35	Fresh		January 2023	0.50	Fresh
	Feb23	0.23	Fresh		February 2023	0.45	Fresh
	Apr23	0.10	Fresh		April 2023	0.10	Fresh

P1: Genésio Dam; P2: Alto da Conceição Bridge; P3: Centro Dam; P4: Santa Luzia Island Canal; P5: East–West Bridge; and P6: Barrocas Dam

Table 2 shows that, at all points studied, the water remained classified as brackish for practically the entire evaluation period, except in the months with higher rainfall, when there was a change to the classification of freshwater, probably due to the dilution process caused by the rains. The present study corroborates the results presented by Bezerra et al. (2013), who classified the water of the same river as brackish in the three dams located in the urban stretch. During the dry season, the concentration of components presents in the water (pollutants, nutrients, or ions) increases, which interferes with the salinity of the water. During drought, the salinity of the water also increases, as a consequence of the low flow rate (Nas & Nas, 2009; Jones & Van Vliet, 2018).

The higher standard deviation and coefficient values (Table 3) may be associated with the spatial heterogeneity of anthropogenic sources, geogenic conditions, and variations in land use. The average EC and Na^+^ values reveal a slight to moderate impact (0.7 dS m^−1^ < EC < 3.0 dS m^−1^) on salinization and plant toxicity, respectively, at the six points studied. Overall, there is more Ca^2+^ than Mg^2+^, presenting a higher Ca/Mg ratio that favors soil structure and permeability, as well as the balance of exchangeable bases in the exchange complex. Furthermore, the average Ca^2+^ and Mg^2+^ contents represent a low to moderate risk of dripper clogging due to carbonate formation. On the other hand, Cl^−^ proved to be the most abundant ion in the river water, indicating that its application by spraying will certainly cause leaf burns, marginal necrosis, and reduced growth in plants, making it necessary to choose more tolerant plants that can limit the translocation of this ion (Capra & Scicolone, 1998; Drechsel et al., 2023). Regarding geogenic conditions, Silva and Camargo (2022) report that the Apodi-Mossoró river basin presents crystalline (igneous and metamorphic rocks) and sedimentary (limestone formations) domains, which directly influence the ionic composition of surface waters, mainly with EC, K^+^ Na^+^, Ca^2+^, Mg^2+^, and Cl^−^. In this study, salinity levels were evaluated using EC values. This attribute allows for the estimation of both salinity and the concentration of total dissolved solids in water (Matos, 2012). Thus, the increase in salt concentration can be intensified by evaporation, mineral leaching, or as a result of human activity, through the discharge of effluents, contributing to increased Cl^−^ levels (Kaushal et al., 2018; Maleki Tirabadi et al., 2021). Therefore, identifying the sources that contribute to salinity is essential for the assessment of water resources (Melesse et al., 2020). The increase in ions at points P1 to P6 can also be attributed to the dams along the river, changes in flow, and the drying up of intermittent rivers during the dry season (Messouli, 2014). The presence of three dams along the urban stretch of the river, combined with its location in the semiarid region of northeastern Brazil, may contribute to the increase in EC and ion levels shown in Table 3.

**Table 3:** Mean (M), median (Me), standard deviation (SD), and coefficient of variation (CV) of salinity-related attributes

Points	Attributes	M	Me	Sd	CV (%)	Points	Attributes	M	Me	Sd	CV (%)
P1	EC	0.96	1.02	0.38	39.33	P4	EC	1.08	1.11	0.50	45.94
	K^+^	0.19	0.16	0.08	42.63		K^+^	0.19	0.17	0.06	31.36
	Na^+^	6.46	6.07	2.82	43.73		Na^+^	7.41	6.65	3.85	51.91
	Ca^2+^	3.89	3.94	1.67	42.96		Ca^2+^	4.36	4.14	1.62	37.15
	Mg^2+^	4.58	4.49	2.84	61.98		Mg^2+^	3.76	3.86	1.76	46.89
	Cl^−^	16.38	13.60	10.43	63.72		Cl^−^	9.18	9.90	5.95	64.87
P2	EC	1.23	1.16	0.33	27.07	P5	EC	1.10	1.21	0.51	45.98
	K^+^	0.19	0.18	0.07	36.77		K^+^	0.15	0.16	0.06	42.82
	Na^+^	6.13	5.97	2.76	45.07		Na^+^	7.61	7.76	3.36	44.17
	Ca^2+^	4.03	4.27	1.66	41.25		Ca^2+^	4.60	4.86	1.63	35.46
	Mg^2+^	2.21	3.01	3.19	144.57		Mg^2+^	3.79	3.95	1.17	30.87
	Cl^−^	21.53	13.50	20.62	95.79		Cl^−^	12.28	12.50	6.36	51.80
P3	EC	0.96	1.08	0.55	57.43	P6	EC	1.02	1.06	0.48	47.21
	K^+^	0.21	0.19	0.06	29.91		K^+^	0.23	0.20	0.08	37.40
	Na^+^	6.90	6.63	3.44	49.85		Na^+^	6.23	7.26	3.45	55.37
	Ca^2+^	3.90	3.94	1.36	34.97		Ca^2+^	4.02	4.21	1.30	32.24
	Mg^2+^	3.01	3.20	1.29	42.85		Mg^2+^	4.20	4.75	1.82	43.45
	Cl^−^	11.20	11.50	4.53	40.43		Cl^−^	11.93	11.90	5.84	48.95

*EC* electrical conductivity in dS m^−1^; K^+^, Ca^2+^, Mg^2+^ e Cl^−^—Potassium, calcium, magnesium and chloride in meq L^−1^; P1: Genésio Dam; P2: Alto da Conceição Bridge; P3: Centro Dam; P4: Santa Luzia Island Canal; P5: East–West Bridge; and P6: Barrocas Dam

Regarding WT, there was a greater temporal than spatial influence on the magnitude of the values (Table 4). Thus, WT showed less variation in relation to the other WQI attributes, with values between 25 and 32 °C. The values from the eight campaigns are in accordance with the standard established by the CONAMA Resolution No. 357/2005 (Brasil, 2005), whose maximum value is 40 °C. This property plays an important role, as it has the capacity to regulate the self-purification of a water body, as well as the DO content in the aquatic ecosystem (Pratiwi et al., 2023).

**Table 4:** Values of the water quality attributes that make up the WQI

Point	Period	WT	pH	TURB	TS	DO	BOD	TC	TKN	Pt
P1	Jul22	25.4	7.78	8.63	557.0	6.96	4.0	2.3	1.505	0.17
	Aug22	28.5	7.36	3.80	790.5	7.98	2.0	0.3	1.893	0.01
	Sep22	29.2	7.72	4.12	891.0	7.59	2.0	0.3	3.494	0.01
	Oct22	28.8	7.78	5.45	1121.7	7.55	5.0	0.3	2.297	0.02
	Dec22	29.3	8.63	7.06	1529.5	10.53	3.0	2.3	1.602	0.02
	Jan23	30.4	8.60	8.78	1636.0	10.3	3.0	0.3	0.582	0.03
	Feb23	31.3	8.60	9.46	1461.3	8.68	4.0	0.3	1.407	0.04
	Apr23	29.0	7.74	16.2	402	3.39	4.0	0.9	1.197	0.04
P2	Jul22	28.0	7.51	66.2	846.5	4.44	5.0	46	5,873	0.01
	Aug22	27.5	7.36	28.4	965.5	3.87	4.0	0.3	1.456	0.05
	Sep22	28.3	7.39	5.35	1073.5	1.61	5.0	2.9	0.437	0.05
	Oct22	28.9	7.46	3.55	1263	1.94	7.0	0.3	2.588	0.02
	Dec22	28.8	7.86	3.51	1554.5	1.52	3.0	15	5.242	0.02
	Jan23	28.5	7.94	3.78	1488	1.67	5.0	1.5	2.475	0.03
	Feb23	30.1	7.81	2.61	1283.3	0.88	3.0	1.5	0.971	0.04
	Apr23	29.9	8.02	48.7	341	5	3.0	2.3	2.896	0.04
P3	Jul22	28.2	7.52	3.01	830	2.25	2.0	9.3	4.562	0.01
	Aug22	28.3	7.26	3.21	1042.5	1.63	1.0	0.3	2.427	0.02
	Sep22	27.8	7.36	9.64	1055.5	3.3	13.0	21	4.659	0.01
	Oct22	28.1	7.43	8.09	1033	1.01	8.0	0.3	3.883	0.01
	Dec22	29.1	7.89	3.91	1396.5	2.87	11.0	110	3.883	0.08
	Jan23	28.1	7.96	4.77	766	3	4.0	110	7.959	0.01
	Feb23	29.9	7.62	8.99	592.7	0.32	4.0	110	1.747	0.15
	Apr23	30.4	8.03	45.00	299	5.53	3.0	24	4.530	0.11
P4	Jul22	28.5	7.59	14.3	776.5	5.42	6.0	4.3	12.036	0.02
	Aug22	27.7	7.47	42.8	1008.5	1.72	16.0	0	7.183	0.07
	Sep22	28.3	7.37	164	1456.5	1.62	19.0	2.3	16.404	0.07
	Oct22	28.7	7.58	2.66	1255.3	2.07	8.0	0.3	11.874	0.05
	Dec22	28.3	7.94	8.26	1495.5	1.04	13.0	1.4	8.057	0.35
	Jan23	28.4	8.02	2.84	1595	3.94	7.0	24	4.077	0.19
	Feb23	29.9	7.95	2.09	1237.7	0.94	3.0	24	1.359	0.15
	Apr23	30.0	8.07	54.1	274	5.1	4.0	2.3	4.497	0.11
P5	Jul22	28.2	7.53	2.22	809	2.8	3.0	0.92	4.271	0.01
	Aug22	28.1	7.38	3.47	918	2.82	5.0	0.3	4.562	0.02
	Sep22	29.3	7.61	8.89	1040.5	4	7.0	0.36	21.841	0.03
	Oct22	29.3	7.87	5.17	1191	5.64	10.0	0.3	10.225	0.01
	Dec22	29.4	8.05	55.6	1437.5	0.5	36.0	24.0	27.567	0.96
	Jan23	29.0	8.07	30.6	1489	3.5	35.0	24.0	12.230	0.21
	Feb23	30.7	8.6	8.47	1140.7	10.11	15.0	24.0	0.582	0.35
	Apr23	29.9	8.07	44	263	5.35	2.0	110.0	13.460	0.27
P6	Jul22	28.5	7.79	4.58	846.0	7.74	3.0	0.72	10.677	0.01
	Aug22	28.0	7.66	6.98	1049.0	5.88	4.0	0.30	2.330	0.03
	Sep22	29.8	7.78	5.94	978.5	5.25	2.0	0.92	6.261	0.02
	Oct22	28.8	8.28	6.62	1127.7	8.79	6.0	0.30	6.423	0.05
	Dec22	28.6	8.69	12.3	1270.5	8.02	6.0	0.92	9.610	0.19
	Jan23	29.7	8.50	28.6	1243.0	6.08	16.0	0.92	7.717	0.18
	Feb23	32.8	9.28	9.35	1129.0	19.15	10.0	2.30	11.891	0.2
	Apr23	30.7	8.04	49.3	320.0	4.36	2.0	15.00	9.739	0.11

*WT* water temperature in °C, *TURB* turbidity in NTU, *NTU* Nephelometric turbidity unit, *TS* total solids in mg L^−1^, *DO* dissolved oxygen in mg L^−1^, *BOD* biochemical oxygen demand in mg L^−1^, *TC* thermotolerant coliforms in MLN 100 mL^−1^, *MPN* most probable number, *TKN* total Kjeldahl nitrogen in mg L^−1^, *Pt* total phosphorus in mg L^−1^

At points P1 to P6, it was observed that the water always remained above 7.0, tending towards an alkaline state, showing a greater influence of time. In aquatic environments, the pH value tends to increase due to the greater activity of photosynthetic algae, which consume carbon dioxide and affect acidity (Khouni et al., 2021). Furthermore, this attribute is influenced by several factors, such as the chemical constituents present in the water, geological formation, and the level of contaminants reaching the water body (Pereira et al., 2022). It is noteworthy that among the main attributes of the WQI, pH is the third most important (wi = 0.12).

Essential for the biota, it is considered a determining variable for the classification of water bodies and is important in altering the classification ranges. pH is a measure that estimates the acidity or alkalinity of water according to the amount of H^+^ and OH^−^ ions available, ranging on a scale from 0 to 14. Therefore, an acidic or alkaline environment can be an indication of anthropogenic or natural pollution Keerthan et al. (2023).

During the dry season, from July to December, when there was little or no rainfall, the TURB (turbidity of water) was very low at all points. With the onset of the rain, the waters were agitated, transporting sediment particles and dragging water from the gutters, significantly increasing the TURB at all points analyzed. However, despite the increase, the values did not exceed CONAMA Resolution No. 357/2005 (Brasil, 2005) threshold of 100 NTU, except for point P4, with 164 NTU, representing a 64% increase. Overall, TURB is characterized by measuring the opacity of water caused by existing particles, whether organic suspensions or metallic precipitates (World Health Organization, 2022). These particles impede the penetration of a beam of light into the aquatic environment, and the intensity of this penetration is measured by the TURB.

Regarding the TS, a spatiotemporal influence on the magnitude of the attribute was observed, to the detriment of surface runoff generated by rainfall, point and diffuse pollution, and geogenic contributions. The lowest TS values occurred in April. Similar TS concentrations were found in a study conducted with water quality indicators in the urban area of Mossoró. In that study, values between 1320 and 1514 mg L^−1^ were found (Bezerra et al., 2013). It should be noted that CONAMA Resolution No. 357/2005 (Brasil, 2005) does not establish a limit for TS. However, TS is an attribute related to the presence of particles being residues present in the water that remain after the evaporation process, allowing the detection of physicochemical changes resulting from effluent discharges and erosive processes. High TS values can cause damage to aquatic life, including fish, as well as causing the retention of bacteria and organic matter in the riverbed. Considered one of the main indicators of pollution caused by effluents, the concentration TS must be estimated so that the necessary adjustments can be made to treatment techniques, especially when the water is intended for human consumption, industrial use, or irrigation (Kyei et al., 2023; Rahman et al., 2021). Several factors can contribute to a large input of TS into surface waters, whether due to the geochemical characteristics of the water body or the discharge of effluents (Silva et al., 2021).

The levels of DO for aquatic preservation are 5 mg L^−1^ (Conselho Nacional do Meio Ambiente, 2005). At all sampling points, at some point during the year, the DO concentration fell below the value established by the CONAMA Resolution No. 357/2005. At points P2, P3, P4, and P5, this value was less than 1.0 mg L^−1^. This drop in DO may have been caused by several factors, such as the disposal of untreated waste, surface runoff, and the use of fertilizers near the riverbed (Bouslah et al., 2017). Low DO concentrations indicate that the water may be polluted by effluents, due to the high content of biodegradable organic matter. Furthermore, DO is a direct reflection of the balance between photosynthesis, respiration, and decomposition (Coffin et al., 2018). Therefore, DO has a very significant weight (wi = 0.17), being the highest among the attributes established for WQI.

BOD is an essential attribute for determining pollution levels in water bodies caused by biodegradable organic matter. The maximum limit established for class 2 freshwater should be a maximum of 5 mg L^−1^ (Brasil, 2005). In the present study, it was observed that BOD fluctuated throughout the entire evaluation period. All points presented, at some point, BOD values above 5 mg L^−1^. The only point that remained within this margin throughout the sampling was P1. On the other hand, points P3, P4, P5, and P6 presented high BOD values at least at some point during the observation, all above 5 mg L^−1^. Studies conducted in reservoirs in the Northeast also found high BOD values, as occurred in 2020 in the Nilo Coelho reservoir in Terra Nova, Pernambuco (Silveira et al., 2022). The Apodi-Mossoró River was analyzed in 2010, and BOD values above 15 mg L^−1^ were also found, demonstrating that the river has been suffering from anthropogenic activity over time, directly impacting the organic load. This reflects the discharge of effluents, in addition to agricultural or livestock activities on its banks (Bezerra et al., 2013). Two parameters show an inversely proportional correlation: BOD and DO. Generally, locations with low DO values present high BOD values due to microbiological activities that increase oxygen consumption (Jonas, 1997). Table 4 shows that this correlation was verified at all points. Point P5 presented the highest BOD value, with 36 mg L^−1^, while it presented very low DO levels, 0.50 mg L^−1^.

The highest population levels of TC occur in December 2022, January 2023, and February 2023 in P3 and in April 2023 in P5, probably due to the discharge of untreated sewage. Regarding TC for primary contact recreation, waters classified as excellent have 80% or more of a set of samples obtained in each of the five previous weeks, collected at the same location, with a maximum of 250 TC, according to CONAMA Resolution No. 274/2000 (Brasil, 2000). It was evident at points P1 to P6 that this condition was met and that the water quality in terms of TC population levels allows for primary contact recreation, such as swimming, water skiing, and diving, considering the resolution. For other water uses, this resolution establishes a maximum of 1000 TC in 80% or more of at least six samples collected during a 1-year period, with a bimonthly frequency, for class 2 freshwater rivers (Brasil, 2005). In terms of microbiological characterization of water, pollution indicators are measured based on the presence of certain organisms, such as viruses, salmonella, protozoa, coliforms, among others (Holcomb & Stewart, 2020). Coliforms belong to a group of bacteria that are excellent microbiological indicators of pollution in surface waters (Mara & Horan, 2003). One class present in this group is that of TC, bacteria that settle in the human intestine and, when excreted, reach water bodies through untreated sewage.

It was noted that TKN levels increased when the river passed through the urban stretch of Mossoró, due to the accumulation imposed by point and diffuse pollution sources. Nitrogen is available in various forms, either reduced or oxidized, such as organic nitrogen, ammonium, ammonia, nitrate, and nitrite, originating from various sources and obtained naturally, such as in the atmosphere, or through human activities. Regarding the WQI assessment, CETESB made an adaptation and began using the total nitrogen value, but in the present study, TKN was characterized. CONAMA Resolution No. 357/2005 (Brasil, 2005) does not establish a standard limit for TKN but describes it in a fragmented way. As for ammoniacal nitrogen, there is a wide range of permitted values, varying from 1.0 to 13.3 mg L^−1^ of N for class 2 freshwater, all fluctuating according to the pH value.

The following reference values are adopted for Pt levels: brackish water rivers class 1, the limit is 0.124 mg L^−1^; freshwater rivers class 2, it is 0.030 mg L^−1^; lentic environments, 0.050 mg L^−1^; intermediate environments, lotic environments, the maximum is 0.1 mg L^−1^ (Brasil, 2005). Due to dams, for most of the year, the Apodi-Mossoró River tends to behave as a lentic environment, which would be the dry season. In the rainy season, it tends to be lotic with or without increased flow. For evaluation, the references for a lentic environment will be used. P1, P4, P5, and P6 showed average and/or maximum Pt concentrations significantly above the permitted level, with P5 registering the highest value, possibly due to the contribution of fertilizers and agricultural residues through surface runoff during the rainy season, given its proximity to a greenhouse that uses these products. Only P3 remained within the reference range, while P2 approached the limit.

The detection of higher nitrogen and phosphorus levels in surface water bodies indicates that the environment receives effluent discharges (Buwono et al., 2021). Nutrients are vital for vegetative growth, with nitrogen and phosphorus considered two of the most important for plant development. However, when present in high concentrations in water bodies, they can be harmful, promoting the eutrophication process, a phenomenon that increases the number of algae, giving the water source a greenish color and preventing the penetration of solar radiation. In addition, during this cycle, many dead algae are degraded by bacteria, which consume oxygen, reducing its availability and causing hypoxia (Pham et al., 2022).

Table 5 shows the WQI classification for the state of São Paulo, according to CETESB (2024a, 2024b), and for the state of Rio Grande do Norte, according to the Agência Nacional de Água e Saneamento Básico (ANA, 2025).

**Table 5:** WQI classification for the states of São Paulo (SP) and Rio Grande do Norte (RN) in Brazil

Points	Period evaluated	WQI	SP	RN	Points	Period evaluated	WQI	SP	RN
P1	Jul22	76	Good	Good	P4	Jul22	77	Good	Good
	Aug22	87	Excellent	Good		Aug22	49	Fair	Poor
	Sep22	86	Excellent	Good		Sep22	38	Fair	Poor
	Oct22	83	Excellent	Good		Oct22	65	Good	Fair
	Dec22	70	Good	Fair		Dec22	48	Fair	Poor
	Jan23	74	Good	Good		Jan23	64	Good	Fair
	Feb23	79	Good	Good		Feb23	49	Fair	Poor
	Apr23	71	Good	Good		Apr23	72	Good	Good
P2	Jul22	60	Good	Fair	P5	Jul22	67	Good	Fair
	Aug22	71	Good	Good		Aug22	66	Good	Fair
	Sep22	58	Good	Fair		Sep22	75	Good	Good
	Oct22	65	Good	Fair		Oct22	83	Excellent	Good
	Dec22	54	Good	Fair		Dec22	25	Poor	Very poor
	Jan23	58	Good	Fair		Jan23	41	Fair	Poor
	Feb23	55	Good	Fair		Feb23	61	Good	Fair
	Apr23	75	Good	Good		Apr23	65	Good	Fair
P3	Jul22	61	Good	Fair	P6	Jul22	83	Excellent	Good
	Aug22	63	Good	Fair		Aug22	82	Excellent	Good
	Sep22	63	Good	Fair		Sep22	79	Good	Good
	Oct22	57	Good	Fair		Oct22	80	Excellent	Good
	Dec22	57	Good	Fair		Dec22	72	Good	Good
	Jan23	61	Good	Fair		Jan23	62	Good	Fair
	Feb23	41	Fair	Poor		Feb23	63	Good	Fair
	Apr23	70	Good	Fair		Apr23	65	Good	Fair

P1: Genésio Dam; P2: Alto da Conceição Bridge; P3: Centro Dam; P4: Santa Luzia Island Canal; P5: East–West Bridge; and P6: Barrocas Dam

At Point 1, which precedes the most urbanized area, the classifications were consistently more positive, ranging from excellent to good according to the São Paulo classification, and predominantly good according to the Rio Grande do Norte standards. This is corroborated by the indicators in Table 4, which show that P1 was the only one to maintain BOD within the limit of 5.0 mg L^−1^ throughout the period, indicating a lower organic load and, consequently, better quality. At points further downstream, such as P2, P3, and P4, there was a tendency towards lower classifications, such as reasonable and even poor (P3 and P4) according to the Rio Grande do Norte classification, especially during the months with lower rainfall. This degradation is closely linked to the increase in pollutants, with BOD values frequently exceeding the limit of 5.0 mg L^−1^ at these points and DO levels sometimes falling below 1.0 mg L^−1^. Point 5 stood out for having gone through all classification ranges, from excellent to poor, with the worst result (WQI of 25) in December 2022. The more pronounced variation in the central and final points (P2 to P6) suggests a strong influence of anthropogenic activities, such as effluent discharge. The Instituto de Gestão de Águas do Rio Grande do Norte (IGARN, 2025), qualifies the CETESB WQI classifications as follows: (1) water suitable for conventional treatment for public supply when the classification is excellent, good, and regular and (2) water unsuitable for conventional treatment for public supply, requiring more advanced treatments for the poor and very poor classifications.

### Normality test and transformation of the attributes that make up the WQI

**Fig. 3:**
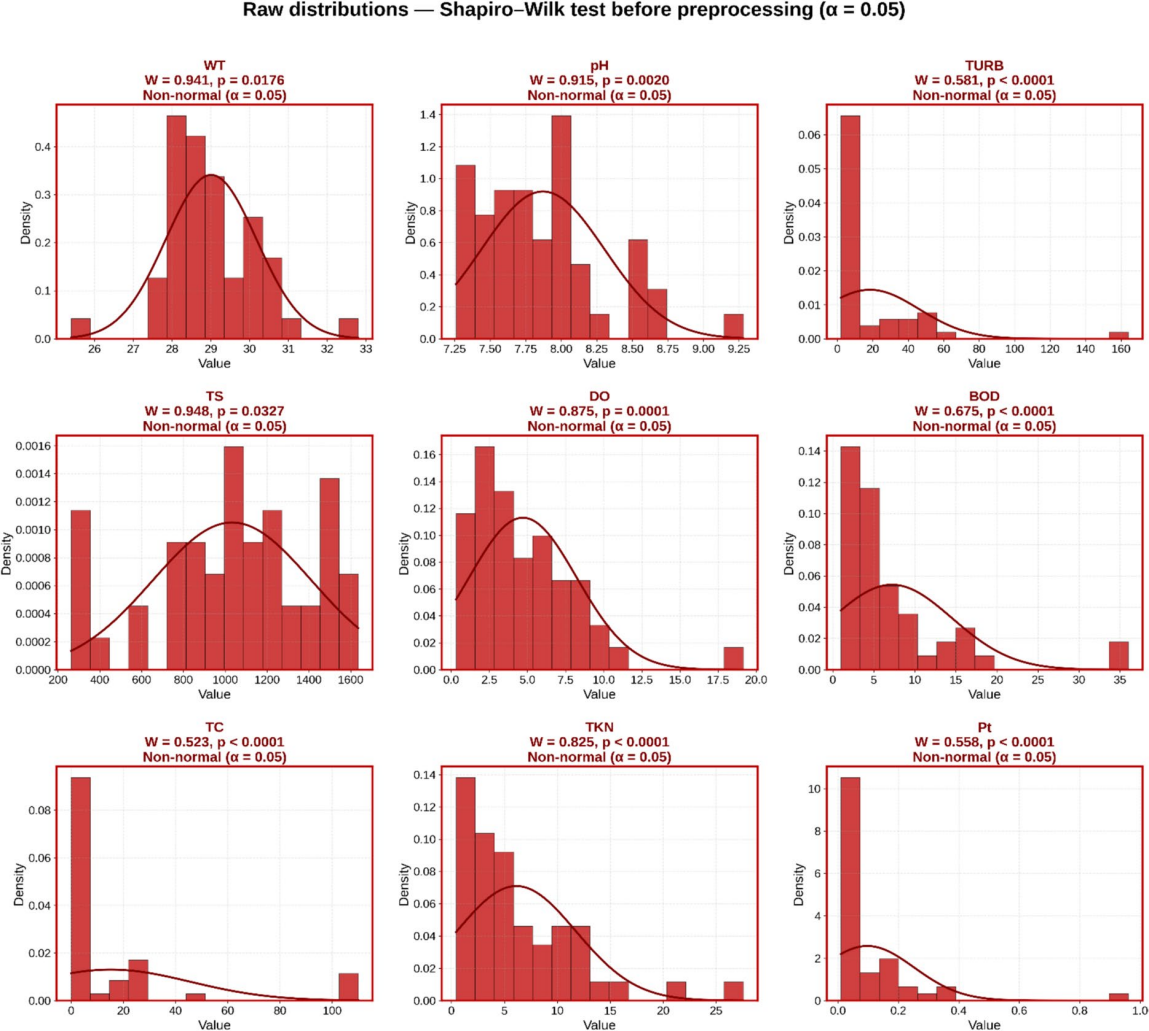
Crude distributions of the nine water quality variables using the Shapiro–Wilk test before preprocessing (*α* = 0.05)

Normality was assessed for each WQI attribute using the Shapiro–Wilk test (*α* = 0.05; recommended for *n* < 50 (Razali & Wah, 2011)) with the raw data. All nine variables showed a significant deviation from normality (*p* < 0.05) (Fig. 3).

**Fig. 4:**
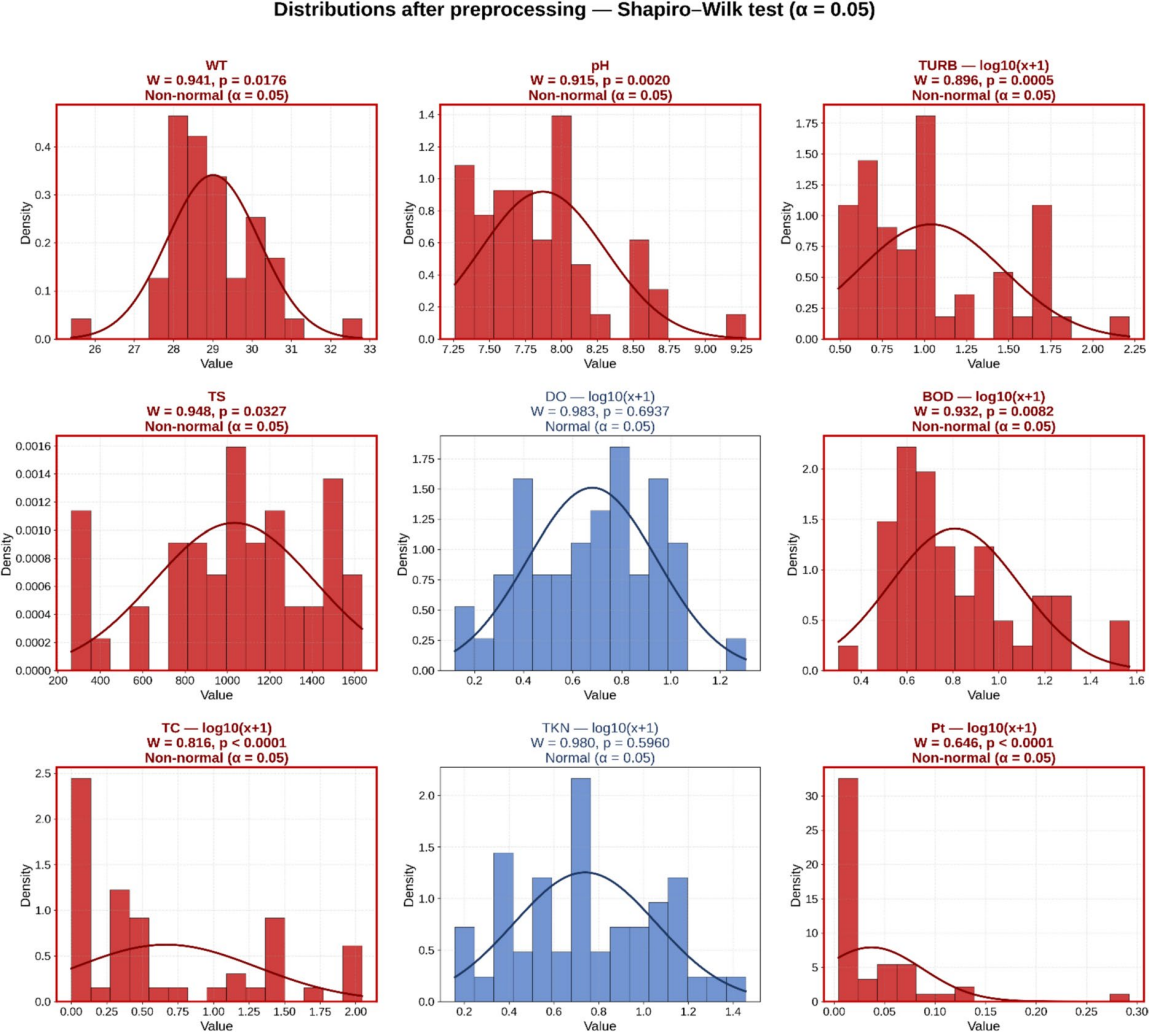
Crude distributions of the nine water quality variables with the Shapiro–Wilk test after preprocessing with log10(*x* + 1) transformation (*α* = 0.05)

To improve the linear structure of the dataset before Pearson-based multivariate analysis, the right-skewed concentration/count variables (TURB, DO, BOD, TC, TKN, Pt) were transformed using log10(x + 1); pH and WT were left untransformed for physicochemical reasons, as pH is already on a logarithmic scale and WT is not a concentration. Preprocessing reduced skewness and improved the distributional behavior of several variables, although not all achieved normality after transformation. DO and TKN became normal (*p* > 0.05), while TURB, BOD, TC, and Pt remained non-normal (Fig. 4).

### Multivariate analysis of the attributes that make up the WQI

#### Heatmap of the Pearson correlation

**Fig. 5:**
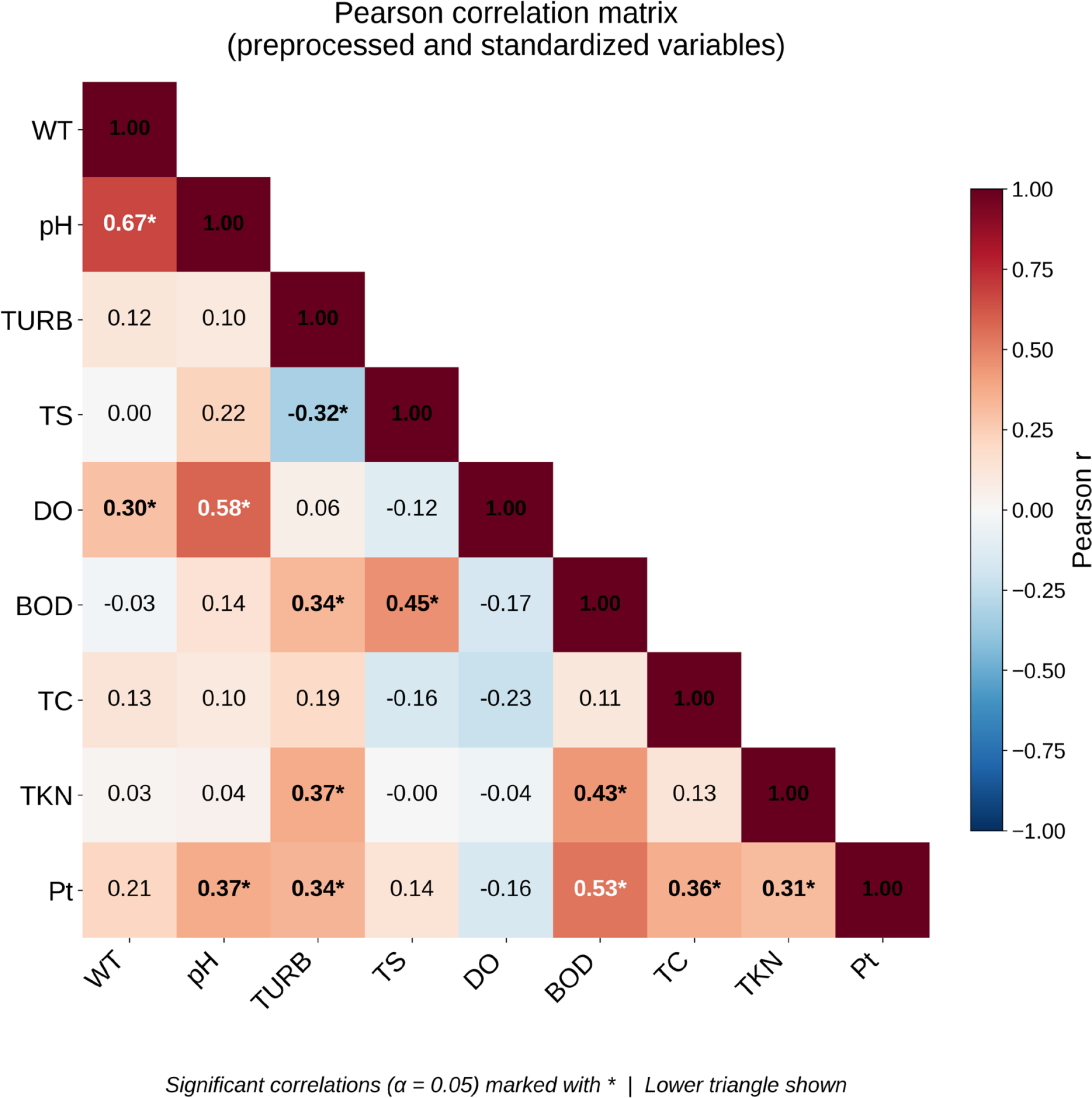
Heatmap of the Pearson correlation matrix between pre-processed water quality variables. Variables skewed to the right were transformed by log10(*x* + 1), and all were standardized to z-score before correlation analysis. Significant correlations (*α* = 0.05) are marked with asterisk (*)

After preprocessing (selective log10(x + 1) transformation and *z*-score standardization), the Pearson correlation matrix shows the following significant linear associations between the nine variables (Fig. 5).

Three sets of associations stand out. First, a strong positive correlation between WT and pH (*r* = 0.67) because temperature affects the autoionization balance of water, altering the concentrations of H^+^ and OH^−^ and, consequently, the pH Keerthan et al. (2023); together with the moderate positive correlations pH–DO (*r* = 0.58) and WT–DO (*r* = 0.30), considering that pH tends to increase in aquatic environments with intense photosynthesis due to CO₂ consumption by algae and macrophytes, while high WT increases the metabolism of organisms and decomposing bacteria, intensifying DO consumption (CETESB, 2024a, 2024b). Second, a network of moderate to strong positive correlations connects the variables associated with organic matter load and nutrient enrichment: BOD–TKN (*r* = 0.43), BOD–Pt (*r* = 0.53), TKN–Pt (*r* = 0.31), TURB–BOD (*r* = 0.34), TURB–TKN (*r* = 0.37), TURB–Pt (*r* = 0.34), and TS–BOD (*r* = 0.45). This clustering suggests a common anthropogenic origin, the discharge of domestic and agro-industrial effluents, which simultaneously increases organic load, nitrogen, phosphorus, and suspended/colloidal material along the urban stretch (Bezerra et al., 2013; Buwono et al., 2021). Third, the moderate positive correlation between TC and Pt (*r* = 0.36) suggests that microbiological contamination co-occurs with phosphorus pulses, consistent with the contribution of untreated sewage. TURB also correlates negatively with TS (*r* = − 0.32), which is compatible with episodes in which suspended (TURB) and dissolved (salinity) loads have distinct dominant origins, surface runoff (diffuse pollution) versus geogenic conditions (CETESB, 2024a, 2024b). These results confirm BOD, TKN, and Pt as the most informative variables to describe the anthropogenic pressure on the Apodi-Mossoró River, and reinforce the role of WT, pH, and DO as the main descriptors of its physicochemical state.

#### Principal component analysis (PCA) and factor analysis (FA) of the attributes that make up WQI

**Fig. 6:**
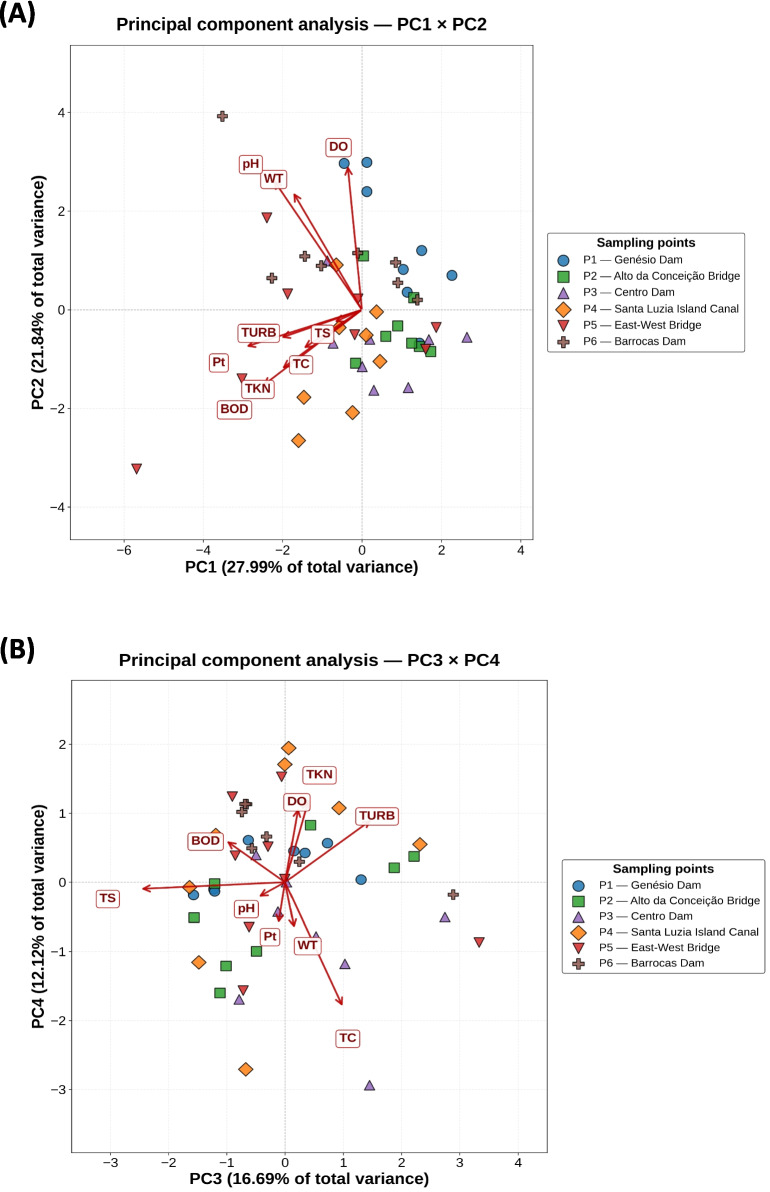
Biplot of the principal component analysis of the WQI attributes: **A** PC1 × PC2 and (**B**) PC3 × PC4

PCA on the correlation matrix of pre-processed and standardized variables retained four components by Kaiser’s criterion (λ ≥ 1), which together explain 78.64% of the total variance (PC1 = 27.99%; PC2 = 21.84%; PC3 = 16.69%; PC4 = 12.12%; see Figs. 6a,b and Table 6). After Varimax rotation with normalization, four interpretable componential clusters emerged. Factor 1 (21.63% of the variance after rotation) groups variables associated with organic matter load and nutrient enrichment: TKN (|λ|= 0.79) and TURB (|λ|= 0.77), BOD (|λ|= 0.68 reached the threshold |λ|≥ 0.65), and Pt also contributes (|λ|= 0.50). There were significant changes in water quality from the first to the last dam. The water quality of the watershed reflects the river's demand and the resulting human activities (Sourn et al., 2022). Evaluating factor 1, higher factor loadings are evident in the attributes TKN, TURB, and BOD, which are indicators of pollution: when present in high concentrations, they directly affect water quality.

**Table 6:** Varimax-rotated factor loadings, eigenvalues, explained variance, and communalities (*h*^2^) for the four components of the standardized WQI attribute Pearson correlation matrix

Variável	Fator 1	Fator 2	Fator 3	Fator 4	Community
WT	0.031	**0.810**	0.044	−0.222	**0.708**
pH	−0.080	**0.934**	−0.189	−0.065	**0.918**
TURB	**−0.768**	0.111	0.375	−0.127	**0.759**
TS	0.067	0.069	**−0.938**	0.074	**0.895**
DO	−0.020	**0.703**	0.230	0.514	**0.811**
BOD	**−0.678**	−0.017	−0.597	−0.145	**0.838**
TC	−0.077	0.054	0.165	**−0.869**	**0.792**
TKN	**−0.791**	−0.034	−0.034	0.001	0.627
Pt	−0.503	0.246	−0.294	−0.572	**0.728**
Eigenvalue	2.519	1.965	1.502	1.091	
% Variance	27.99	21.84	16.69	12.12	
Cumulative %	27.99	49.83	66.52	78.64	

Retention by Kaiser criterion (λ ≥ 1); Factor loadings with |λ|≥ 0.65 highlighted in bold; Bartlett's test of sphericity: χ^2^ = 150.388, *p* < 0.001; and KMO (Kaiser–Meyer–Olkin) overall = 0.48

With the increase in organic load, a very high bacterial population level is necessary to degrade this organic matter, which requires high oxygen consumption, resulting in reduced levels of water oxygenation. Water oxygenation levels are extremely important information for the assessment of natural waters and the possible impacts of pollution and eutrophication (Jordão et al., 2007).

Factor 2 (23.38%) captures the physicochemical gradient: pH (|λ|= 0.93), WT (|λ|= 0.81), and DO (|λ|= 0.70). The factors DO, TEMP, and pH increased together positively in factor 2, in opposition to BOD, TKN, and TC. In locations with good oxygenation, BOD levels were low, as were nitrogen and microorganism levels, indicating low anthropogenic activity and eutrophication. pH is influenced by the oxidation of organic matter, CO₂ concentration levels, and temperature (Reis et al., 2025). Temperature influences solubility and chemical reactions in water, also directly affecting other attributes such as BOD and DO. The variation in WT was contrary to that of BOD and did not negatively influence the DO level, when the opposite should normally have occurred. This was probably a consequence of the interference of other factors, such as salinity and altitude. The level of DO tends to decrease with increasing WT, since oxygen solubility decreases. Water temperature is a determining factor in estimating the DO concentration in a body of water (Khanna et al., 2012). Considering that DO is influenced not only by temperature, but also by nitrogen and phosphorus, which have a large impact because they increase nutrient concentration, promoting the growth of algae and populations of photosynthetic cyanobacteria. During the day, these organisms perform photosynthesis, saturating the environment and releasing oxygen. At a point with probable eutrophication, the DO level was found to exceed 19 mg L^−1^. It is understood that pH had a significant influence on the DO level in the water. This occurs because a higher pH favors the growth of aquatic plants. These plants, through the process of photosynthesis, release oxygen into the water, which can cause oxygen saturation in the aquatic system. The growth of aquatic plants increases photosynthetic activity, releasing oxygen into the environment (Kim et al., 2003).

Factor 3 (17.59%) is dominated by TS (|λ|= 0.94), representing the dimension of suspended solids/dissolved solids that represent the salinity of the river hydrochemistry in a semiarid region. TS constitutes an important indicator of water quality in rivers, as it reflects the presence of suspended and dissolved materials capable of altering transparency, light penetration, ecological balance, and water treatment processes for human use. Its concentrations can be influenced by anthropogenic sources, such as sewage discharge, industrial effluents, agricultural activities, and urban runoff, as well as by geogenic sources associated with rock weathering, natural soil erosion, sediment transport, and natural input of mineral salts, especially in semiarid regions with high evaporation (Bezerra et al., 2013; CETESB 2024a, 2024b).

Factor 4 (16.04%) is dominated by TC (|λ|= 0.87) and represents microbiological contamination, with Pt also contributing (|λ|= 0.57). TC and Pt show a strong correlation in river waters, as both are associated with sewage discharge and anthropogenic pollution, simultaneously indicating fecal contamination and nutrient enrichment in the water body that results in eutrophication (Yuan et al., 2019).

Communalities exceed 0.7 for WT, pH, TURB, TS, DO, BOD, and TC, indicating that more than 70% of the variance of each of these variables is captured by the four-component solution; TKN (*h*^2^ = 0.63) and Pt (*h*^2^ = 0.73) are also adequately represented. Bartlett’s test of sphericity (*χ*^2^ = 150.39; *p* < 0.001) supports the existence of a relevant correlation structure; however, the overall KMO of 0.48 indicates that the rotated solution should be interpreted with caution and that PCA should be considered the main multivariate outcome.

The results showed that the study hypothesis was accepted, as there was a spatiotemporal influence on river quality as a function of dry and rainy periods, where factors such as dilution, evapoconcentration, geogenic contribution, and point and diffuse pollution governed the magnitude of the physical, chemical, and microbiological attributes analyzed. Furthermore, it was found that the worst WQI classifications occur in the urban stretches most affected by anthropogenic pollution. Future studies recommend a continued and broader evaluation in terms of water pollution indicators, including emerging contaminants.

## Conclusions

There was high spatial and temporal variability in the indicators studied, influenced by the local climate, which has concentrated rainfall and an extended dry season throughout the year, and by human activity, evidenced by urbanization.

The river water showed a high content of dissolved salts, especially Na^+^ and Ca^2+^, resulting in a classification as brackish at all points for most of the year, being considered fresh after the rainy season. Due to the variability of salt content, influenced by climatic factors, the Apodi-Mossoró River fluctuated between class 1 brackish rivers and class 2 freshwater rivers, a variation that makes it impossible to definitively classify it.

Water Quality varied considerably, covering all classifications from excellent to inadequate. The best water quality was observed at the point located before the urbanized area, where the WQI showed ratings from excellent to good throughout the year, indicating less influence from anthropic activities.

The Barrocas Dam presented all forms of classification. At the height of the drought in December, this point reached its worst moment, being classified as poor, influenced by pollution resulting from greater urbanization.

In the multivariate analysis of WQI attributes, TKN, TURB and BOD were the variables that most influenced the classification and are most related to anthropogenic activity.

## Data Availability

All data generated or analyzed during this study are included in this published article.
